# Functional Analysis of Jasmonates in Rice through Mutant Approaches

**DOI:** 10.3390/plants5010015

**Published:** 2016-03-18

**Authors:** Rohit Dhakarey, Preshobha Kodackattumannil Peethambaran, Michael Riemann

**Affiliations:** Botanical Institute, Karlsruhe Institute of Technology, Kaiserstr. 2, 76131 Karlsruhe, Germany; rohitbiotech@yahoo.co.in (R.D.); preshobhakp@gmail.com (P.K.P.)

**Keywords:** jasmonate, rice, *Oryza sativa*, mutant, photomorphogenesis, abiotic stress, biotic stress, development

## Abstract

Jasmonic acid, one of the major plant hormones, is, unlike other hormones, a lipid-derived compound that is synthesized from the fatty acid linolenic acid. It has been studied intensively in many plant species including *Arabidopsis thaliana*, in which most of the enzymes participating in its biosynthesis were characterized. In the past 15 years, mutants and transgenic plants affected in the jasmonate pathway became available in rice and facilitate studies on the functions of this hormone in an important crop. Those functions are partially conserved compared to other plant species, and include roles in fertility, response to mechanical wounding and defense against herbivores. However, new and surprising functions have also been uncovered by mutant approaches, such as a close link between light perception and the jasmonate pathway. This was not only useful to show a phenomenon that is unique to rice but also helped to establish this role in plant species where such links are less obvious. This review aims to provide an overview of currently available rice mutants and transgenic plants in the jasmonate pathway and highlights some selected roles of jasmonate in this species, such as photomorphogenesis, and abiotic and biotic stress.

## 1. Introduction

Rice (*Oryza sativa* L.) is the staple food for around half of the world’s population. Therefore, providing a stable yield from this crop is not only crucial for economic reasons, but also necessary to provide social and political stability, especially in large areas of Asia where nutrition of the population is almost entirely based on rice. In order to understand mechanisms of rice growth, development and its response to environmental cues, the elucidation of regulatory mechanisms involving plant growth regulators, commonly known as plant hormones, is very important. In fact, fundamental results have been obtained for some plant hormones in rice related research for the first time in history. For example, gibberellic acid has been identified from exudates of the fungus *Gibberella fujikuroi* as the causal agent of the bakanae disease of rice [1]. Intriguingly, recently, another milestone was formed in gibberellic acid research by identification of its receptor GID1 in rice [2]. Rice was used with regard to research about other plant hormones as well. A well-known example in plant physiology is the rice lamina inclination assay, a bioassay that can be used to quantify the concentration of active brassinosteroids in plant extracts [3].

**Figure 1:**
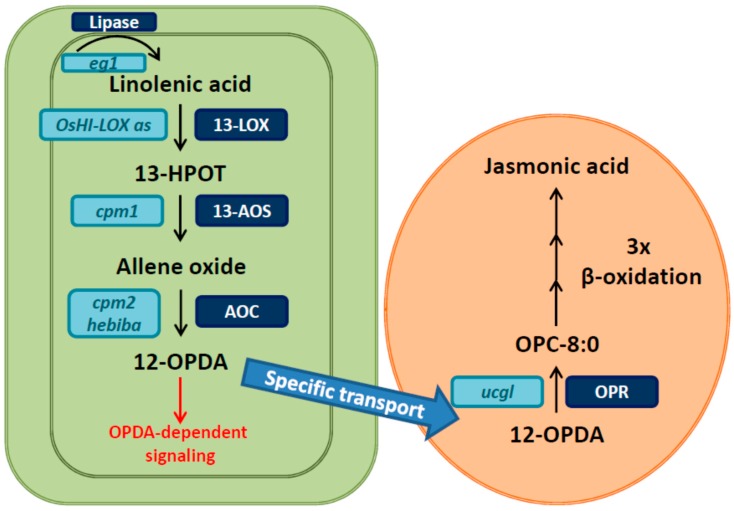
Biosynthesis of JA, major enzymes involved and mutants in the pathway. The biosynthesis occurs in chloroplasts (green) and peroxisomes (brown). In brief, after cleavage of linolenic acid from a membrane lipid it is converted to OPDA in three enzymatic steps. OPDA is a functional signaling compound but can be transported to peroxisomes specifically where it is further metabolized to JA by the action of OPR and subsequent β-oxidation steps. For further explanation, refer to the text. Mutant names are shown in light blue boxes. *Extra glume 1* (*eg1*) is a mutant of a plastidic lipase involved in flower specific JA synthesis. *OsHI-LOX* antisense plants were found to be impaired in JA-dependent insect responses. A mutant of *OsAOS1*, *coleoptile photomorphogenesis 1* (*cpm1*), was isolated as photomorphogenic rice mutant like *hebiba* and *cpm2*, which are mutated in *OsAOC*. OsOPR7 carries a point-mutation in *unclosed glumes* (*ucgl*). Abbreviations: 13-LOX: 13-lipoxygenase, 13-HPOT: (13S)-hydroperoxyoctadecatrienoic, 13-AOS: 13-allene oxide synthase, AOC: allene oxide cyclase, 12-OPDA: 12-oxo-phytodienoic acid, OPR: OPDA reductase, OPC-8:0: 3-oxo-2(2’(Z)-pentenyl)-cyclopentane-1-octanoic acid.

Jasmonic acid (JA), a plant hormone synthesized from the fatty acid linolenic acid, is an important plant growth regulator with versatile functions in development and in the response to environmental challenges. Despite of its various roles, and especially its roles in plant stress responses, knowledge in rice has been small compared to other plant species such as *Arabidopsis thaliana*, tomato or tobacco [4]. Its biosynthesis and signaling pathways have been elucidated since the 1980s comprehensively, which was reviewed by Wasternack and Hause [5] on a large scale. JA biosynthesis takes place in two compartments, the chloroplast and the peroxisomes (Figure 1). It is initiated by a lipase that cleaves linolenic acid from a membrane lipid of the chloroplast membrane. Linolenic acid can serve as a substrate for either 9- or 13-LIPOXYGENASES (LOX), but 13-LOXs are required for the biosynthesis of JA. ALLENE OXIDE SYNTHASE (AOS), and ALLENE OXIDE CYCLASE (AOC) convert the product of 13-LOX, (13S)-hydroperoxyoctadecatrienoic acid (13-HPOT), which results in the formation of the intermediate 12-oxo-phytodienoic acid (OPDA). This compound has signaling activity by itself [6]; however, for the synthesis of JA, it is transported from the chloroplast to the peroxisomes, where it is reduced by an enzyme called OPDA REDUCTASE (OPR) and subsequently goes through several steps of β-oxidation to shorten the side chain. The final product in the peroxisomes is JA, which can freely move to the cytosol.

**Figure 2:**
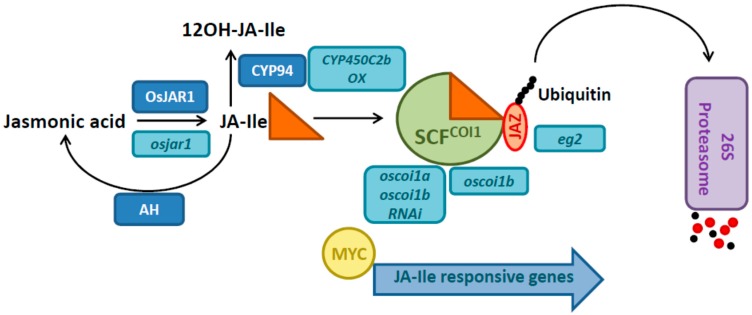
Activation, inactivation, perception and signaling of JA. Names of mutants and transgenic lines are shown in light blue boxes. JAR1 catalyzes the conjugation of JA to isoleucine (JA-Ile). Several alleles of rice *jar1* mutants are available because it is a hotspot of Tos17 retrotransposon insertion. Further GH3 enzymes may contribute to the biosynthesis of JA-Ile in rice. JA-Ile is recognized by its receptor COI1, which functions as E3 ubiquitin ligase in a SCF complex. Subsequently, JAZ proteins are recognized by the hormone receptor complex, poly-ubiquitinated and degraded in the 26S proteasome. *Extra glume 2 (eg2)* is a mutant of *OsJAZ1*. MYC transcription factors are released from repression by JAZ proteins and can activate transcription of early response genes. JA-Ile can be inactivated by CYP94 enzymes or amidohydrolases. In transgenic approaches, signaling has been affected by overexpressing CYP84C2b. Abbreviations: JA-Ile: jasmonoyl-isoleucine, JAR1: JASMONATE RESISTANT 1, COI1: CORONATINE INSENSITIVE 1, JAZ: JASMONATE ZIM-domain, CYP94: CYTOCHROME P450 CYP94 subfamily, AH: AMIDOHYDROLASES.

JA itself is presumably not an active signaling compound, and needs to be conjugated to the amino acid isoleucine (Ile) in a reaction catalyzed by the GH3 enzyme JASMONATE RESISTANT 1 (JAR1) to initiate signaling [7]. JA-Ile is recognized by its receptor CORONATINE INSENSITIVE 1 (COI1) [8], an F-box protein forming an SCF complex, which operates as an E3 ubiquitin ligase (Figure 2). The hormone receptor complex recruits JAZ proteins, repressors of JA signaling, and catalyzes their poly-ubiquitination, which marks them for proteolytic degradation in the 26S proteasome [9,10]. After that, MYC transcription factors are relieved from repression by JAZ proteins, and activate the transcription of early JA responsive genes, amongst which are transcripts of the JAZ repressors and further transcription factors. Recently, it has also become obvious that not just the synthesis of JA-Ile, but also the inactivation of JA-Ile is a possibility to adjust JA responses. Two major mechanisms to metabolize JA-Ile have been described: one operating through CYTOCHROME P450s (CYP94 family) [11,12], and another one through AMIDOHYDROLASES such as IAR3 and ILL6 in *Arabidopsis* [13].

Many regulatory functions in the life cycle of plants as diverse as leaf senescence [14], sex determination [15], tendril coiling [16], or photomorphogenesis [17] have been assigned to JA. Amongst those, some are very obviously relevant for applied purposes in an agricultural crop, e.g., fertility, wounding and defense [5]. Therefore, the mechanisms of JA biosynthesis and signaling that are particular for rice or other crops have to be studied in the respective plant species, even when the major architecture of the pathways is conserved. A key for functional analysis is the availability of plants altered in the expression of specific genes. In the past decade, considerable progress has been achieved in the field, which will facilitate future studies focusing on the role of JA for rice biology, and also strengthen the basic knowledge necessary for targeted strategies to improve important agricultural traits. In this review, we provide an overview of such genetic materials, and show that some novel findings were reported using those materials.

## 2. Rice Mutants and Transgenic Plants in the Jasmonate Pathway

Although many rice mutant lines are publicly available, the coverage of the whole genome is not as good as in *Arabidopsis.* In the JA pathway, however, mutants in a few key enzymes have been identified or plants impaired in the function of specific genes in the pathway have been generated by transgenic approaches (Figure 1). Such materials are available for both the biosynthesis and the signaling pathway. In the biosynthesis pathway, there is one step that is encoded by a single copy gene, which is the conversion of allene oxide to OPDA by AOC. Enzymes in other steps of biosynthesis are encoded at least by two homologues, e.g., *AOS* exists in two copies [18,19]. Therefore, rice mutants of *AOC* are convenient and reliable JA deficient genotypes, because no redundantly active enzymes could compensate for its function.

Two *aoc* mutants, *hebiba* and *coleoptile photomorphogenesis 2* (*cpm2*), have been identified [20,21], and can serve as genetic material free of enzymatically produced JA. Both mutants have been generated by γ-ray irradiation, however, while *hebiba* has a deletion of approximately 170 kbp, *cpm2* has a deletion of 11 bp in the first exon of the *AOC* gene [21,22]. Therefore, *cpm2* can be considered as an *AOC*- specific mutant while for *hebiba* additional mutations have to be considered, including a receptor for karrikins [23]. However, many phenotypes were found to be similar in both mutants, including photomorphogenesis, fertility, early flowering, flower architecture, response to salt stress, and susceptibility to *Magnaporthe oryzae.* Hence, these phenotypes can be directly related to the deficiency in JA in both mutants. As there is no other source of active JA, it can also be concluded that these physiological functions depend on JA in rice. For AOS, the enzyme upstream of AOC, a mutant called *cpm1* has been published previously [24]. In *cpm1*, the gene encoding for OsAOS1 carries a point mutation leading to an amino acid exchange in the protein, and to strongly reduced enzymatic activity of the enzyme *in vitro* [18,25]. Hence, *cpm1* might still be able to synthesize some JA for two reasons: (1) OsAOS1 might still be partially active in the mutant plant; and (2) another AOS (OsAOS2, [26]) might have redundant function. Beside these plastid localized enzymes, a third enzyme in the same compartment, 13-LOX, has been targeted by transgenic approaches. Silencing of *OsHI-LOX* led to decreased levels of JA in leaves and roots upon herbivore chewing in shoots and roots [27,28]. This LOX might be specific for defense responses of rice against herbivores as no other phenotypes have been reported for those lines.

One of the prominent functions of JA is in reproductive development. A very obvious phenotypes of *hebiba* is its male sterility [20], which is due to the inability to open the anthers. But other flower related phenotypes have been recognized in *hebiba* and *cpm2*, including morphological changes and alteration of heading time [21]. In mutant approaches other JA-related genes have been functionally assigned to flower related phenotypes: *extra glume 1* (*eg1*) was identified to be a mutant of a chloroplastic lipase involved in flower-specific biosynthesis of JA [29], *unclosed glumes* (*ucgl*) [30] was found to carry a point mutation in *OsOPR7*, encoding the enzyme catalyzing the first step of JA biosynthesis in the peroxisomes [31], and mutants of *OsJAR1* have been shown to be defective in husk closure and anther dehiscence [32,33]. Furthermore, *EXTRA GLUME 2* (*EG2*), encoding for OsJAZ1, a repressor of JA signaling, was isolated as a mutant affected in spikelet development [29]. Different from *AOC*, all these genes are not single copy genes in rice, and therefore redundancy might hamper mutant approaches. Furthermore, some of the mutants mentioned, e.g., *cpm1* or *ucgl* [24,30], carry point mutations in biosynthetic genes, and therefore a partially functional enzyme might be expressed.

To date, the only known receptor of JA, perceiving JA-Ile, in *Arabidopsis* is COI1 [8,9,10]. Rice has three F-box proteins that are close orthologs of COI1 designated as OsCOI1a, OsCOI1b and OsCOI2 [34]. It has been demonstrated that 2 of them, OsCOI1a and OsCOI1b, are able to complement the *coi1* mutant of *Arabidopsis* [35]. Both genes were knocked down simultaneously in RNAi lines, leading to enhanced growth and limitation in defense capability [36]. Furthermore a *oscoi1b* knockout mutant showed delayed leaf yellowing indicating its function in senescence [37]. The presence of three closely related F-box proteins with high homology to COI1 suggests that perception mechanisms in rice may differ from *Arabidopsis*, and that it might have more possibilities of distinguishing different jasmonate members on the level of perception. There are 15 *JAZ* genes in rice [38], and for some of them functional analysis has been carried out (Table 1). Overexpression of *OsJAZ9* led to an increased salt tolerance [38], while its suppression reduced salt tolerance [39], supporting a function of JA signaling in abiotic stress tolerance (see Section 5 and Table 1). *OsJAZ8* was found as a JA-inducible gene [40]. In the same study it was shown that overexpression of a truncated version of *OsJAZ8*, lacking the C-terminus, partially impaired defense against *Xanthomonas oryzae*. With regard to *JAZ* genes also developmental functions were reported. Overexpression of *OsJAZ10* caused increase in shoot growth and seed size [41], and *OsJAZ1* (*eg2*) mutants showed an altered floral development [29]. Certainly more functions will be assigned to specific rice *JAZ* genes in the near future as the molecular signaling mechanism is also currently under investigation.

## 3. Jasmonates and Their Link to Light

First JA biosynthesis mutants were isolated due to their altered growth response to red light perceived by the phytochrome (phy) photoreceptors [17,20,24]. It could be shown that biosynthesis of JA is induced in response to red light in rice coleoptiles, the coleoptile phenotype of *hebiba* could be complemented by exogenous addition of MeJA [20]. It was shown as well that a considerable number of red light inducible genes in the coleoptile are JA-dependent [18,25,42]. Later it was proven that the gene responsible for the coleoptile phenotype of *hebiba* and *cpm2* mutants is the JA biosynthesis gene *AOC* by genetic complementation with the wild type *AOC* gene [21]. The fact that JA is more prominent during the photomorphogenic response of rice coleoptiles as compared to *Arabidopsis* hypocotyls might be due to the principal difference of the tissues involved: rice coleoptiles are protective organs which elongate quickly in darkness (under the soil) to allow the young leaves to reach the surface and light soon. Perception of light is a signal to stop elongation, open and eventually the coleoptile is degenerating after a few days. The function of JA might be not merely related to growth inhibition but also to senescence in coleoptiles. This is different in hypocotyls of dicotyledonous plants in which the hypocotyl is not degenerating after perception of light. However, the link between light and JA is clearly established in *Arabidopsis* as shown by several studies [43,44,45,46,47]. The link between light and JA has also been intensively studied with regard to modulation of defense responses depending on light conditions, which has been reviewed by Kazan [48].

Evidence suggests that JA is not only a signaling molecule downstream of phy as it has been shown that one of the three phys in rice, phyA, which normally is quickly degraded in red light, is more stable in *hebiba* [49,50]. Hence, JA not only transduces the light signal but has an impact on the perception of light through regulation of the amount of photoreceptors in the tissue as well. Another interesting regulatory mechanism was revealed in a study using *phyAphyC* double mutants which were exposed to blue light [51]. Blue light led to a clear reduction of coleoptile length in the wild type and the mutant, however the mutant coleoptiles were significantly longer. Despite the reduced growth inhibition, the mutants accumulated more JA and JA-Ile, suggesting that phyA might regulate the activation or inactivation of JA. In a related study, mutants of the JA-Ile conjugating enzyme OsJAR1 were examined. This study showed that JA-Ile levels can still be induced in rice *jar1* mutant coleoptile in response to light irradiation to wild type levels, while mutant leaves, which were mechanically wounded, were impaired in JA-Ile synthesis. Thus, JA-Ile is not the only active jasmonate in light-mediated coleoptile growth inhibition, because despite of the normal JA-Ile levels *osjar1* mutants display a clear photomorphogenic phenotype [32,52]. Another GH3 enzyme in rice, designated as OsJAR2, has been shown to be able to conjugate JA and Ile [53], and additional amino acids [52] *in vitro*; hence, this enzyme might complement for OsJAR1 in rice *jar1* mutants. However, evidence for this assumption is lacking and warrants further investigations. Those should include investigation of other JA amino acid conjugates and regulation of JA inactivation pathways.

## 4. Jasmonates and Their Role in Rice Defense

JA has been shown to be important for the interaction with other organisms and is well-known for its function as a hormone involved in defense responses [54,55]. In rice several reports have been published showing a function for JA in the defense against insects [27,28,56,57,58]. However, they are also important for the defense against some microorganisms. One of the economically important diseases in rice agriculture, rice blast, is caused by the hemibiotrophic fungus *Magnaporthe oryzae*, which during its life cycle goes through necrotrophic and biotrophic stages [59]. Especially the penetration of the host cell by the appressorium can be considered as a severe mechanical wounding event [60], to which plants would likely respond by induction of JA. Evidence for a function of JA in the defense against this fungus has been found earlier. JA-dependent genes were upregulated during *Magnaporthe* infection [61,62]. Overexpression of *OsAOS2* caused an enhanced resistance against the blast fungus [26].

**Figure 3:**
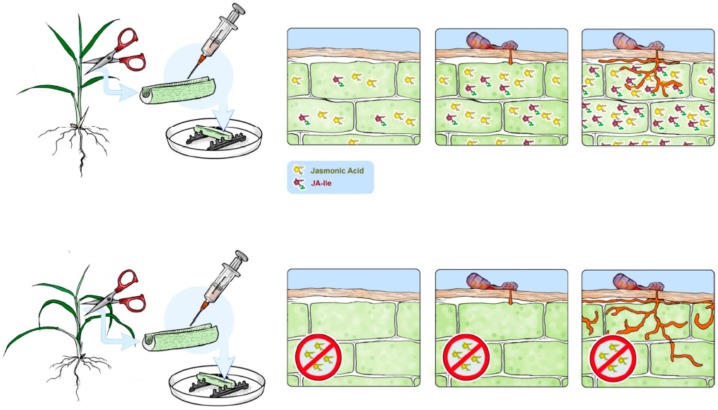
Function of JA in incompatible interaction of rice and *Magnaporthe oryzae*. Leaf sheath segments of rice plants were inoculated with an incompatible strain of *Magnaporthe oryzae*. The wild type (WT) produces JA and JA-Ile, probably in response to appressorium penetration. Dependent on the production of JA-Ile, the plant produces phytoalexins such as sakuranetin, momilactones and phytocassanes. The mutant *cpm2* and *hebiba*, which are impaired in JA biosynthesis, do not accumulate sakuranetin and less momilactones. Correlating with lower JA-Ile and sakuranetin levels, fungal hyphae spread more easily in the mutants.

However, how does JA contribute to the defense against *Magnaporthe* in wild type plants? Recent studies using *aoc* (*hebiba* and *cpm2*) and *jar1* mutants in comparison to their respective wild types have shown that these mutants do not show a difference in susceptibility to compatible, but to incompatible strains of *Magnaporthe* [21,63]. Rice plants produce phytoalexins deriving from different metabolic pathways in response to blast infection [64]. In leaf sheath segment assays, it was found that the wild type, which is able to produce JA and JA-Ile, is also able to produce phytoalexin such as sakuranetin momilactones, and phytocassanes. The accumulation of sakuranetin, a flavonoid phytoalexin [65], strictly depended on JA, because *hebiba* and *cpm2* did not accumulate this compound, while momilactone accumulation was only partially affected and phytocassanes were accumulated like in the wild type. Correlating with JA and sakuranetin deficiency fungal hyphae could spread better in the mutants. Based on these findings it could be postulated that the wild type is able to activate its defense against incompatible *Magnaporthe* strains, because JA biosynthesis can be activated locally at the site of appressorium penetration (Figure 3). Compatible strains might have evolved strategies to inactivate JA-dependent defense mechanisms in rice. In accordance with this notion, such a mechanism has been described recently for a fungal enzyme hydroxylating JA to inactive 12OH-JA [66]. In future, cytological studies are required in order to understand where in the plant tissue biosynthesis and signaling of JA takes place, or is suppressed by the pathogen.

The interaction of salicylic acid (SA) and JA pathways in defense responses are important for host–pathogen and plant–insect interactions. For rice, this has been reviewed by De Vleesschauwer *et al.* [55] and Okada *et al.* [34] in detail. Recently several groups have shown the involvement of WRKY transcription factors in defense responses of rice [67,68,69,70,71,72], which could serve as a hub for JA- and SA-dependent signaling. In the future, it will be required to understand how these are linked to the respective hormonal pathways during defense responses.

## 5. Jasmonates and Their Relevance for the Response to Abiotic Stress

Jasmonates are intensively studied with regard to abiotic stress, including drought and salinity stress [73]. However, the information is sometimes contradictory at first glance. For example, JAs have been reported to accumulate in response to salinity stress in tomato or rice [74,75]. Moreover, a salt tolerant cultivar of rice shows higher endogenous JA contents as compared with a salt-sensitive cultivar, as well as the observation that exogenous MeJA can reduce the uptake of sodium in this salt-tolerant cultivar [76], indicates a function for JAs in salt adaptation. However, it is difficult to draw general connection between high levels of JA and adaptation as it was found in a study about two grapevine cell lines with different salt tolerance levels that JA accumulation was more in the sensitive *Vitis riparia* than the salt tolerant *Vitis rupestris* [77]. The contradictory results suggest that it is not the presence and absence of JA that decides salinity response, but the right timing and control [73,78].

Previously, abiotic stress tolerance has been linked with JA in a report showing enhanced stress tolerance of plants overexpressing JAZ repressor proteins [38]. Recently the function of JA in the response to high salinity has been addressed in two different approaches: *aoc* mutants and transgenic plants overexpressing genes encoding for JA metabolizing CYP450s [79,80]. Under salt stress the *aoc* mutants *hebiba* and *cpm2* displayed reduced salt sensitivity in their phenotype showing longer roots and less severe necrotic leaves. The shoots of the mutants accumulated significantly (by 25%–30%) less sodium ions and showed high chlorophyll content than the wild type. However, there was no significant difference between the mutants and the wild type in sodium contents in the roots, this could mean either that uptake of sodium through the non-selective cation channels is reduced, and/or that the extrusion of sodium through the SOS1 exporter is promoted [81]. When the oxidative events and the antioxidative system of wild type and mutant was analyzed, it was reported that the salinity-induced level of malondialdehyde in wild type shoots was ~50% higher compared with *cpm2* and almost twice that observed in *hebiba*, and also the wild type accumulated ~60% more H_2_O_2_ compared with the mutants. It was also found that two genes induced by oxidative stress were not induced as strongly in the JA biosynthesis mutants, indicating that the mutants were experiencing less oxidative stress or better capable of buffering it. In this study a significant increase in OPDA was found in the wild type, but JA and JA-Ile showed no change under the conditions tested. Mainly based on research results from *Arabidopsis*, OPDA is also discussed as one of the highly reactive electrophile species (RES) responsible for signaling in chloroplasts [82]. OPDA can induce retrograde signaling when bound to its putative receptor cyclophilin 20-3 [83]. When the receptor-hormone complex interacts with serine acetyltransferase, it stabilizes the formation of cysteine synthase and hence the redox homeostasis in plastids is altered. A similar mechanism could exist in rice. As *aoc* mutants are impaired in the biosynthesis of JA and OPDA, this signaling pathway would not be active in the mutants, and this may also be advantageous for the adaptation of rice to salt stress.

Another study reported that the enhanced expression of the CYP450 family gene CYP94C2b confers salt stress tolerance in rice. JA-Ile is deactivated in *Arabidopsis* by conversion into 12OHJA- Ile and 12COOH-JA-Ile, mediated by two closely related CYP450 enzymes, CYP94B3 and CYP94C1 [11,12,84]. The levels of JA-Ile after wounding and responses to exogenous JA decreased in lines overexpressing CYP94C2b. Decrease in JA content can also be correlated with lower expression of JA responsive genes (*JAmyb* and *JAZ11*). This shows the partial suppression of JA response in CYP94C2b overexpressing plants compared to the wild type. When grown under various saline conditions like hydroponic culture and soil cultivation, the transgenic line showed enhanced viability. Their shoots were 80% viable compared to 10% in wild type under high salt concentration and could recover and regain its fertility when transferred to soil. It was proved that plant viability of the overexpressors under saline condition may be associated with preservation of functional shoot meristems, leaf primordia and also delayed progression of leaf senescence. Under normal conditions the number of senescent leaves remained unchanged, which suggests that endogenous JA may play only a limited role in senescence without salinity stress. Similarly, it was reported that *Arabidopsis* LOX2 RNAi lines, in which JA biosynthesis was disrupted, sorbitol stress-induced senescence was delayed but not the natural senescence [85,86]. In contrast, leaf senescence is delayed in the maize *opr7opr8* double mutant, which is deficient in JA production [87].

Comparing results which were found in *aoc* mutants and CP94C2b overexpressing plants, it can be concluded that decreased amounts of JA correlates with an improved performance on high concentrations of NaCl. However, deeper investigations are required in order to understand how jasmonates are involved in the salinity response in the wild type.

Interestingly, another study identified drought as a stress signal that alters jasmonate signature by stopping the conversion of OPDA to JA and further reported that OPDA is the functional convergence point of oxylipin and ABA pathways in order to control stomatal aperture in plant-adaptive responses to drought stress [88]. They used three *Arabidopsis thaliana* ecotypes to examine the oxylipin signature in response to specific stresses, and determined that wounding and drought differentially alter oxylipin profiles, particularly the AOS branch of the oxylipin pathway, which is responsible for production of JA and its precursor OPDA. Specifically, wounding induced both OPDA and JA levels, whereas drought induced only the precursor OPDA. Levels of the phytohormone abscisic acid (ABA) were also mainly enhanced by drought and little by wounding. To explore more about the role of OPDA in plant drought responses, they also generated a range of transgenic lines and harnessed the existing mutant plants that differ in their levels of stress-inducible OPDA but display similar ABA levels. The plants which, were producing higher OPDA levels, exhibited enhanced drought tolerance and reduced stomatal aperture.

In rice, it has been previously reported that there is a correlation between grain yield and MeJA level [29,89]. By overexpressing *Arabidopsis* jasmonic acid carboxyl methyltransferase (*AtJMT*) in rice, the levels of MeJA increased, which in turn resulted in a noticeable decline in the grain yield. Interestingly the decline in yield in the transgenic plants was similar to decline in yield of wild type plants which were exposed to drought stress [89]. It was suggested that, during the early stages of rice flower development under drought condition, production of MeJA is promoted by the induction of OsJMT1, a rice ortholog of AtJMT. Furthermore, it was postulated that the increased accumulation of MeJA in turn activates the expression of OsSDR, which resulted in the increased level of ABA biosynthesis. ABA is also accumulated during drought stress directly, independently from MeJA. As a result, the accumulation of both ABA and MeJA affects spikelet development and subsequently results in reduction in grain yield.

Despite the classical assignation of specific roles to each hormone, it is nowadays widely accepted view that the expression of multiple genes and flux of various metabolic pathways must be systemized in order to adjust the plant response to the severity of the stress, in a time- and tissue-specific manner. The systematically coordinated action of different signaling pathways allows a plastic hierarchy of cross-coordination which is just beginning to be understood [90]. ABA and JA signaling pathways can interact together at several points and there exists an overlap between ABA- and JA-induced gene expression and physiological processes [91]. Water stress may trigger the accumulation of ABA, which is considered to be the key phytohormone involved in regulating whole plant responses to this specific kind of abiotic stress condition. ABA acts in regulatory role in many adaptive plant responses such as inducing the genes responsible for osmotic adjustment, root hydraulic conductivity, root and shoot growth, organ abscission and transpiration [92,93,94]. The ABA downstream signaling in response to water stress is well characterized but the initial switch connecting the primary sensation of water stress and the induction of ABA biosynthesis is still elusive. On the other hand, JA levels are rapidly and transiently increased by turgor reduction induced by water deficit [95,96]. Jasmonate mediated signaling in response to dehydration has been suggested to exist [97]. However, like in salinity response in rice, deeper studies are required in order to show which are the active jasmonates in drought response, as evidence for different players exists [88,98].

## 6. Conclusions

Plants respond to their environment through several well-defined mechanisms but the traits associated with response mechanisms are multigenic—often converging on genes shared by signals. Often, the biological significance as well as the mechanism of crosstalk happening between different signaling pathways operating still remains unsettled. However, with the advent of new tools, these pathways are getting better resolved, which allows the exploration of the physiological, genetic, and biochemical basis of such processes. In this era, the genomic, proteomic and metabolomic approach is now becoming widely used in rice like in other model species. Hopefully with the evolution of these investigative strategies, new cross talks will be unraveled that are active between several different classes of hormones including JA.

Mutant approaches have been extremely useful to elucidate the function of JA in different physiological and developmental contexts and will also be needed to fully understand its involvement in the above-mentioned signaling network. It can be examined in the future using available and new mutants. Novel strategies for targeted genetic engineering will accelerate the functional analysis of genes for which no mutants are available yet. A big challenge will be whether it is possible to make use of deeper knowledge of JA function in future breeding programs.

## Figures and Tables

**Table 1:** Functional assignment to specific *OsJAZ* genes. Identification (ID) number according to MSU and RAP are indicated.

Gene Name	RAP ID	MSU ID	Function	Reference
*OsJAZ1, OsTIFY3*	Os04g0653000	LOC_Os04g55920	Floral development	[29]
*OsJAZ8, OsTIFY10c*	Os09g0439200	LOC_Os09g26780	Plant defense	[40]
*OsJAZ9, OsTIFY11a*	Os03g0180800	LOC_Os03g08310	Salinity stress	[38,39]
*OsJAZ10, OsTIFY11b*	Os03g0181100	LOC_Os03g08330	Stem growth and grain size	[41]
